# One-Pot Facile Green Synthesis of Silver Nanoparticles Using Seed Extract of* Phoenix dactylifera* and Their Bactericidal Potential against MRSA

**DOI:** 10.1155/2018/1860280

**Published:** 2018-06-26

**Authors:** Mohammad Azam Ansari, Mohammad A. Alzohairy

**Affiliations:** ^1^Department of Epidemic Diseases Research, Institute of Research and Medical Consultations (IRMC), Imam Abdulrahman Bin Faisal University, P.O. Box 1982, Dammam 31441, Saudi Arabia; ^2^Department of Medical Laboratories, College of Applied Medical Sciences, Qassim University, Saudi Arabia

## Abstract

Due to the great economic, health, and medicinal importance,* Phoenix dactylifera* seeds were chosen for the synthesis of silver nanoparticles (AgNPs) because of their ecofriendly, nonhazardous, cost effectiveness advancement over physical and chemical methods, as green methods are safe, one step, and simple and did not require any chemical reducing and stabilizing agents. The green synthesized AgNPs were characterized by UV-Vis spectroscopy, SEM, HR-TEM, and DLS. Further, the bactericidal activity of synthesized AgNPs against Methicillin-resistant* Staphylococcus aureus* (MRSA) was investigated by determining MIC/MBC, agar diffusion methods, and electron microscopy. TEM images of the so-formed AgNPs revealed that the NPs were spherical in shape, with a size range of 14–30 nm. The MIC and MBC of AgNPs for MRSA were found to be 10.67±0.94 and 17.33±1.89 *μ*g/ml, respectively. The antibacterial activities were found to be increased with the increasing concentration of AgNPs. The zone of inhibition was greater (24mm) at highest concentrations (500*μ*g/ml) of AgNPs, while smaller (11mm) at lowest concentrations (7.8*μ*g/ml). The SEM images of treated MRSA cells showed wrinkled and damaged cell wall, indicating the disruption and disorganization of membrane. HR-TEM analysis exhibits extensive injury and complete disintegration of cell wall and membrane. Large translucent zones have been seen in the cytoplasm, due to either localized or complete separation of the cell membrane from the cell wall. Overall, these results indicate that green synthesized AgNPs should be considered as an effective treatment and prevention option for the medical devises related infections caused by deadly MRSA and other drug resistant pathogens.

## 1. Introduction

Among noble metal nanoparticles, silver nanoparticles (AgNPs) gain great interest due to their broad applications in medicine, dentistry, drug delivery, tissue and tumour imaging, biolabeling, biosensing, optics, coating for solar energy absorption, catalysis, mirrors, photography, electronics, and the food industry [1–3]. The antibacterial, antifungal, antiviral, and antiparasitic activity of AgNPs is well documented in the literature [4–11]. AgNPs can be synthesized by a large numbers of physical, chemical, biological, and hybrid methods. The comparison between the advantages of green routes of synthesis of AgNPs by* Phoenix dactylifera *seed extract with the disadvantages of AgNPs synthesized by physical, chemical, and biological methods was shown in Table 1. The nanoparticles synthesized from chemical and physical methods generally require high temperature, pressure, expensive equipment, toxic chemicals, and reagents and most importantly capping agents for the stabilization of nanoparticles; thus, these methods are toxic to environment and nonecofriendly. The green routes of synthesis for AgNPs have additional advantages such as ecofriendliness, cost effectiveness, inexpensive, safer, and fast and provide natural capping and stabilization agents. The green synthesis of AgNPs is based on the mechanisms of plant extract assisted reduction of metal salt due to the presence of phytochemicals such as terpenoids, flavonoids, phenolic and dihydric phenols, aldehydes, carboxylic acids, and enzymes, i.e., hydrogenases and reductases [2, 3].

In the present study, we discuss the green synthesis of AgNPs using the commercially economic and abundantly available* Phoenix dactylifera* seed extract as bioreductant and stabilizer. For decades, natural medicinal plant products are used as precious remedies to treat a large number of diseases as they are economically inexpensive and easy to access.* Phoenix dactylifera* is one of the most important and major economic food and crops of the Arab world. The fruit seeds contain a large number of nutritionally important functional compounds, e.g., fatty acids, sugars, protein, fibers, ash, minerals, and vitamins as well as high amounts of phenolic and flavonoids [12, 13]. Date seeds are one of the major waste materials that constitute about 6.1–11.5% of the fruit [14]. Date seeds have antioxidant and free radical scavenging activity as they contain considerable amounts of alkaloids, flavonoids, anthraquinone, saponin, terpenoids, and tannin [15]. The date seeds are generally used as animal feed and are also potential sources of edible oils and pharmaceuticals. Date seeds are often used in alternative and folk medicine for the management of diabetes, hypertension, cancer, liver diseases, gastrointestinal, and cardiovascular disorders and also used to improve the functionality and integrity of the immune system [13, 15, 16]. Moderate antibacterial properties of acetone and ethanolic extract of date seed have also been reported against* Bacillus cereus*,* Staphylococcus aureus, Enterococcus faecalis, Methicillin-resistant Staphylococcus aureus, Pseudomonas aeruginosa, *and* Escherichia coli *[17–19]. The aim of present work was (i) the biosynthesis of AgNPs using date seed extract (DSE) as bioreductant and stabilizing agents; (ii) characterization of green synthesized AgNPs by various sophisticated techniques; (iii) evaluation of antibacterial potential of synthesized nanoparticles against Methicillin-resistant* Staphylococcus aureus *(MRSA ATCC 43300) using twofold serial dilution and agar well diffusion methods; and (iv) characterization of morphological and ultrastructural changes caused by AgNPs on MRSA.

## 2. Materials and Methods

### 2.1. Materials

Silver nitrate (AgNO_3_) with ≥99.5% purity was purchased from Merck, Germany. Mueller Hinton Agar, Luria Bertani broth, and other culture media were obtained from Sigma-Aldrich chemical Co. (St. Louis, MO, USA). Methicillin-resistant* Staphylococcus aureus *(MRSA ATCC 43300) was used for the antibacterial experiments.

### 2.2. Collection of Date Seeds and Aqueous Extracts Preparation

*Phoenix dactylifera* (date palm) seeds were collected from main campus of Qassim University, Al-Qassim, Saudi Arabia. The surface of date seeds was disinfected using 30% sodium hypochlorite for 5 min and rinsed with sterile distilled water several times. In the next step, the seeds were placed in 70% alcohol for 2 min and then washed three times with sterile distilled water. 10 g of seeds was milled using an ordinary grinder and ground kernel was boiled with 100 ml distilled water at 80°C for 20 min. The ground mixture was centrifuged and then the solution was filtered by 0.45 *μ*m Millipore membrane filter and followed by 0.2 *μ*m Millipore membrane filter. The filtrate extract was stored at 4°C and used as reducing and stabilizer agent for the synthesis of silver nanoparticles [20].

### 2.3. Date Seed Extract Mediated Synthesis of Silver Nanoparticles (DSE-AgNPs)

In a typical reaction procedure, 10 ml of seed extract was added to 90 ml of 10^−3^ (M) aqueous silver nitrate solution. The flask (aqueous) was then incubated at room temperature for overnight. Any colour changes of the solution were observed.

### 2.4. Characterization of AgNPs

*UV-Vis Spectroscopy.* The formation and stability AgNPs were carried out by measuring the UV-visible spectra of the solutions after diluting the sample. Distilled water was used as a blank solution. The absorbance spectra of AgNPs solution were recorded at wavelength ranging from 200 to 800 nm by UV-Vis spectrophotometer (Varian Inc., USA).

### 2.5. Scanning and Transmission Electron Microscopy

The morphological features of synthesized DSE-AgNPs were characterized by SEM (Carl Zeiss EVO 40, Germany) with accelerating voltage of 20 kV. AgNPs were sonicated for 10 min before being used. The shape and size of AgNPs were characterized by higher resolution transmission electron microscope (HR-TEM). For HR-TEM, a drop of dispersed solution was placed on a copper grid at room temperature. The HR-TEM images were obtained using a Tecnai G2 (FEI, Electron Optics, USA) transmission electron microscopy with an accelerated voltage of 200 kV.

### 2.6. Dynamic Light Scattering (DLS)

The hydrodynamic size of the AgNPs was determined by DLS as described by Jalal et al. [20]. DLS is commonly used to determine the size distribution or average sizes of synthesized AgNPs in the suspensions.

### 2.7. Methods for Characterization of Antibacterial Activity of DSE-AgNPs

*Determination of Minimal Inhibitory Concentration (MIC).* The MIC of was determined in Luria Bertani broth using twofold serial dilution of DSE-AgNPs as previously described [4]. The MIC is the lowest concentration of AgNPs that completely visually inhibits the 99% growth of the bacteria.

*Determination of Minimal Bactericidal Concentration (MBC).* The MBC is defined as the lowest concentration of AgNPs that kills 100% of the initial bacterial population. The MBC was determined on MHA plates as previously described [20].

### 2.8. Determination of Antibacterial of DSE-AgNPs by Agar Well Diffusion Method

Zone of inhibition test was performed on MHA plates supplemented with 7.8, 15.6, 30.25, 62.5, 125, 250, and 500 *μ*g/ml of AgNPs as described previously with slight modification [21]. Briefly, 20 ml of MHA was poured in well rinsed, autoclaved Petri plates, then 1.0 ml of active bacterial culture was homogeneously spread on the agar plates, and 100 *μ*l of AgNPs solution has been filled in deep blocks, prepared by cutting the agar by gel puncture. The plates were incubated at 37°C for 24 h. The zone size was determined by measuring the radius of the zone in millimetres [21].

### 2.9. Action of DSE-AgNPs on the Morphology of S. aureus as Examined by SEM

The effects of DSE-AgNPs on the morphology of S. aureus were carried out using SEM as described by Ansari et al. [22]. Briefly, the cells (10^6^ CFU/ml) treated with different concentrations of AgNPs were incubated at 37°C for 24 h and then centrifuged at 12000rpm for 10 min. Cells without AgNPs have been taken as control. The pellets were washed with phosphate buffered saline (PBS) and prefixed with 2.5% glutaraldehyde for 1 h at 4°C. The prefixed cells were washed with PBS and postfixed with 1% osmium tetroxide for 1 h at 25°C. After washing with PBS, dehydration process was done with 30, 50, 70, 80, 90, and 100% of acetone (each for 10 minutes). The cell biomass was fixed on the aluminium stubs and then dried overnight in a critical point dryer, sputter-coated with a thin layer of gold (Sputter coater- Polaron SC7640). The coated samples were observed under scanning electron microscope (EVO 40, Germany) with accelerating voltage of 20 kV.

### 2.10. Action and Visualization of Effects of DSE-AgNPs on* S. aureus* as Examined by TEM

To study the nanoparticles-bacterial interaction and their internalization inside the bacterium, the cells treated with different concentration of AgNPs were incubated in LB broth for 16 h. Control experiment was carried out without nanoparticles. After 16 h, treated and untreated cells were washed phosphate buffer and fixed with 2.5% glutaraldehyde for 1 h at 4°C. The prefixed cells were again washed and postfixed in 1% osmium tetroxide and were washed again and dehydrated with a series of graded acetone and then embedded in white resin overnight to polymerize [22]. Ultrathin sections were made with a microtome diamond knife and then stained with uranyl acetate and counterstained with 4% lead citrate. The sections were mounted on copper grids and finally, the internal structure was examined by high-resolution transmission electron microscopy (TEM, Electron Optics, USA) at 120KeV.

## 3. Results and Discussion

### 3.1. Visual Observation and UV-Vis Spectrophotometer Analysis

**Figure 1:**
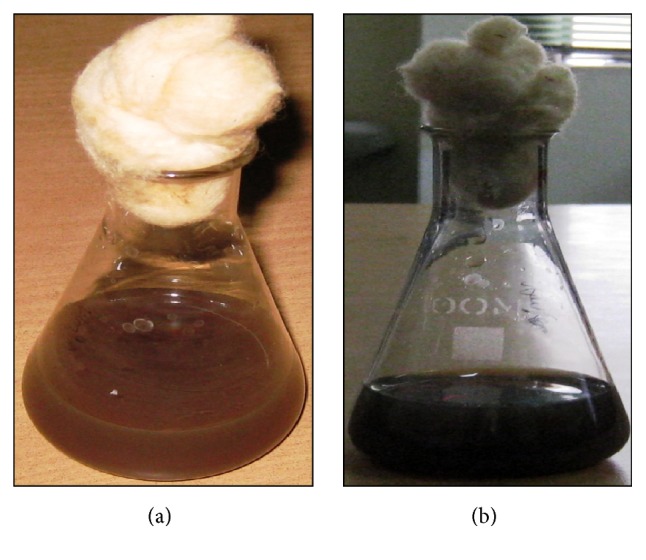
Aqueous solution of 10^−3^ M AgNO_3_ with* Phoenix dactylifera* seed extract. (a) Before adding the seed extract and (b) after addition of seed extract.

**Figure 2:**
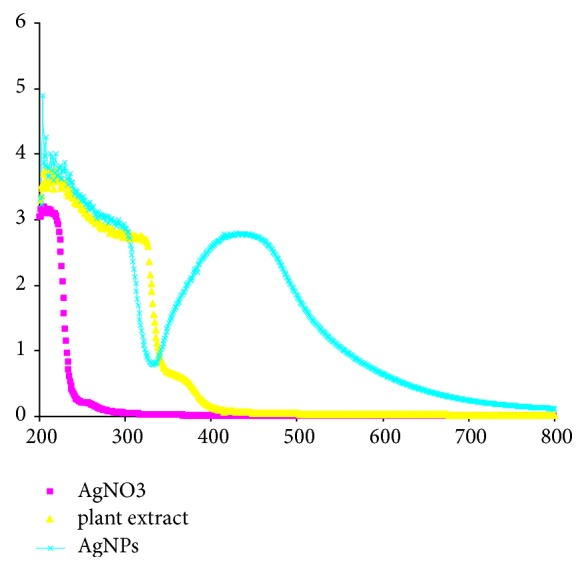
UV-visible spectra of aqueous solution of 10^−3^ M AgNO3 with the* Phoenix dactylifera* seed extract.

The potential of plant extracts to reduce metal ions into metal nanoparticles has been documented in the literature [6, 23]. The aqueous date seed extract (DSE) has the ability to reduce the silver nitrate salt into AgNPs and further they stabilized the synthesized AgNPs due to presence of bioactive phytochemicals in the date seed extract such as phenolics, flavonoids, polyphenols, aldehydes, carboxylic acids, anthraquinone, saponin, terpenoids tannin, and proteins [2, 3, 15]. On adding the aqueous seed extract of date to the silver nitrate solution, a colour change from pale yellow to dark brown has been observed (Figure 1). The appearance of brown colour was primarily due to the excitation of surface plasmon vibrations and this colour change is an indicator of synthesis of AgNPs [24, 25]. Bioreduction of silver ions to metal nanoparticles using various plants parts and change in colour has also been observed by several authors [6, 23]. UV-Vis spectroscopy is an important technique to examine the formation of metal NPs in aqueous solution. The formation of AgNPs in aqueous solution was monitored by measuring the absorption spectra at a wavelength range of 200 to 800 nm; a single, strong, and broad surface plasmon resonance (SPR) peak was observed at 429 nm throughout the reaction period suggesting that the NPs were dispersed in the aqueous solution (Figure 2). AgNPs are known to display a UV-Vis absorption spectrum maximum in the range of 400–500 nm due to the plasmon resonance displayed by AgNPs [26, 27]. These findings showed the similarity with the results reported by other researchers in past [23, 27, 28], who used different plants parts for the synthesis of AgNPs.

### 3.2. SEM and TEM Analysis of Synthesized AgNPs

**Figure 3:**
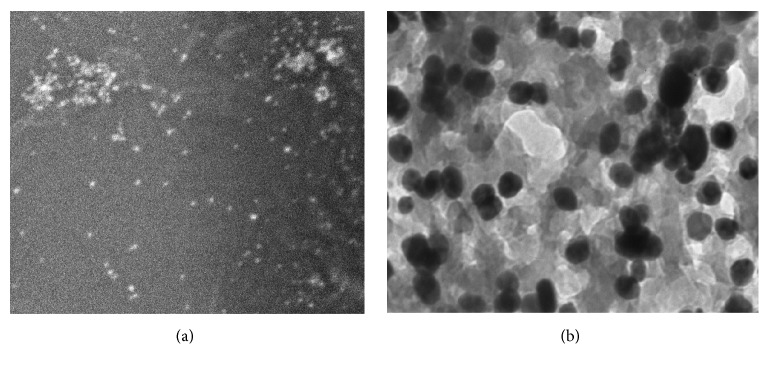
SEM (a) and TEM (b) image of biosynthesized AgNPs using aqueous seed extract of* Phoenix dactylifera*.

In order to determine the morphology and size detail of AgNPs synthesized by date seed extract, scanning and transmission electron microscopy was carried out. Scanning electron microscopy was used to examine the morphological structure of the green synthesized AgNPs (Figure 3(a)). TEM is one of the most powerful tools which can give direct structural and size information of the nanoparticles. Thus, the detailed shape and size of AgNPs were carried out with TEM. The TEM images of the so-formed AgNPs revealed that the NPs were spherical in shape, with a size range of 14–30 nm, in which few NPs were agglomerated (Figure 3(b)). At higher magnification, the TEM micrograph revealed that AgNPs were not in physical contact but separated by uniform distance with some deviations. The capping of AgNPs has also been observed under TEM micrograph (Figure 3(b)). This capping might be because of presence of phytochemicals compounds present in extract [29].

### 3.3. DLS Measurement

**Figure 4:**
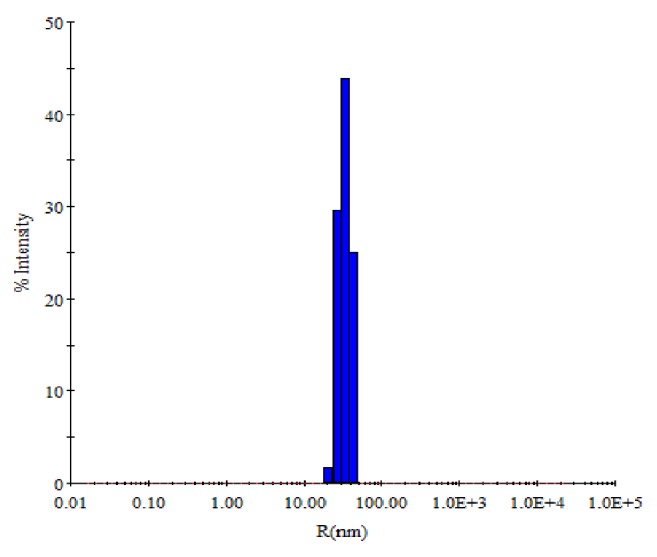
DLS histogram of green synthesized AgNPs.

The TEM measurements were done under high vacuum conditions that require a dry sample; therefore, complimentary experiments were performed to obtain mean size of nanoparticles in aqueous solutions using Dynamic light scattering. DLS is a sophisticated and most frequently used technique to determine the size distribution and average diameter of particles in suspension which utilizes the illumination of a suspension of particles or molecules undergoing Brownian motion by a laser beam [30]. The size distribution histogram of DLS indicates that the average diameter of synthesized AgNPs was 32.1 nm (Figure 4), confirming the results obtained by HR-TEM (Figure 3(b)).

### 3.4. Antibacterial Activity of DSE-AgNPs

**Figure 5:**
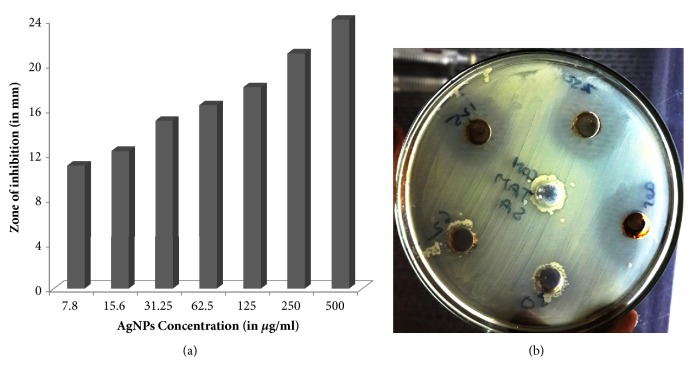
Images of antibacterial activities of synthesized AgNPs against MRSA.

The antibacterial potential of green synthesized AgNPs was investigated against Methicillin-resistant* S. aureus* (ATCC 43300) using twofold serial dilution and agar well diffusion methods (Figure 5). The MIC and MBC values of AgNPs for Methicillin-resistant* S. aureus* (ATCC 43300) were found to be 10.67±0.94 and 17.33±1.89 *μ*g/ml, respectively. Our findings are in agreement with previous studies of Das et al. [31], who demonstrated that MIC and MBC values for AgNPs synthesized from leaf extract of* Ocimum gratissimum* against MRSA were 8 and 16 *μ*g/ml, respectively. Our data on MIC and MBC indicate better antibacterial activity as compared to the previous work of Ayala-Nuez et al. [32] in terms of the MIC and MBC values of AgNPs against MRSA i.e., 1800 *μ*g/ml and 2700 *μ*g/ml, respectively. The antibacterial activity of AgNPs was further examined by agar well diffusion method. The zone of inhibition (in mm) around the well was shown in (Figure 5(b)). It was observed that the antibacterial activities were found to be increased with the increasing concentration of AgNPs (Figure 5(a)). The zones of inhibition were found to be larger (24 mm) at highest concentrations (500 *μ*g/ml) of AgNPs, whereas zone of inhibition was smaller (11 mm) at lowest concentrations (7.8 *μ*g/ml) of AgNPs (Figure 5(a)). These results are in agreement with previous work that examined the antibacterial activity of AgNPs synthesized by using extract of different parts of the plant [31, 33].

### 3.5. Cell Integrity Disruption of Bacteria Induced by AgNPs: Electron Microscopic Analysis

**Figure 6:**
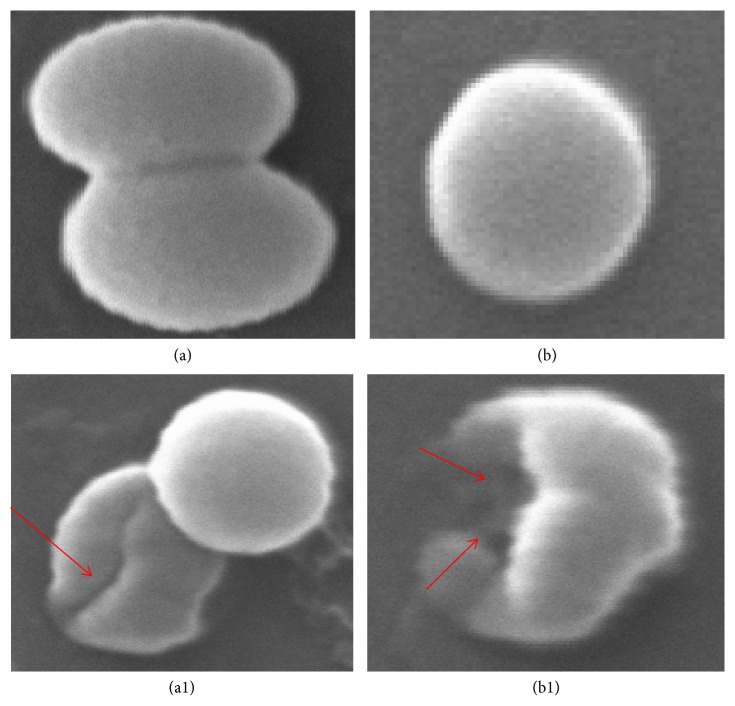
SEM micrograph of MRSA. (a and b) Untreated control cells. ((a1) and (b1)) Cells treated with 25 and 50 *μ*g/ml of AgNPs; red arrows illustrating structural deformities and irregular cell surface.

**Figure 7:**
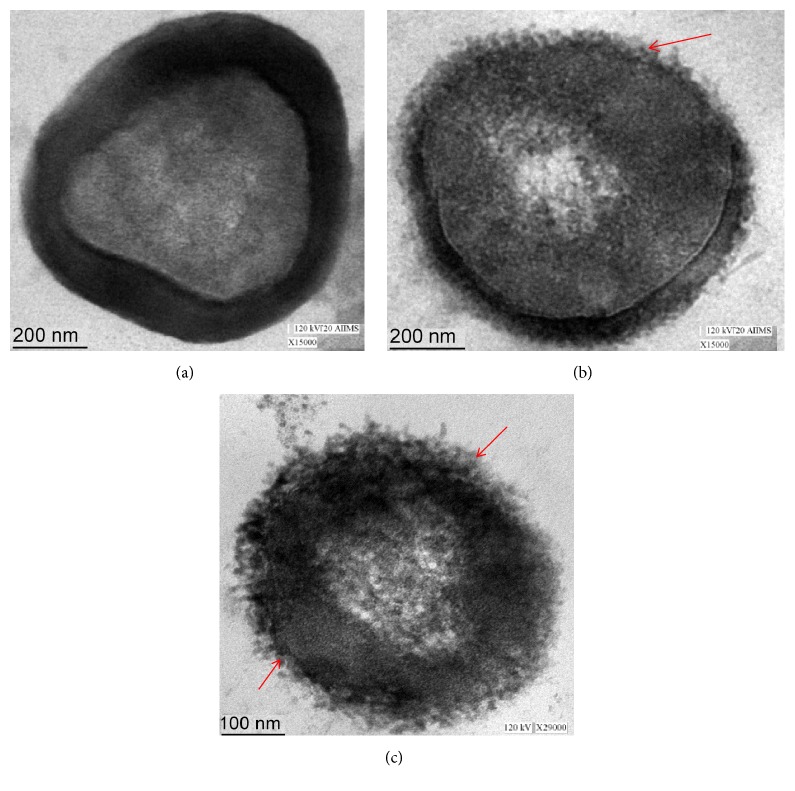
HR-TEM micrograph of MRSA: (a) Untreated control cell; (b and c) treated with 25 and 50 *μ*g/ml AgNPs. Red arrows indicate the attachment and penetration of NPs and degradation and destruction of the outer most layers of cell wall and cytoplasmic membrane.

The MIC/MBC and well diffusion methods clearly demonstrate that AgNPs effectively inhibit the growth of MRSA. Therefore, we investigated the morphological and ultrastructural changes in MRSA cells before and after exposure to AgNPs by scanning electron microscope (SEM) and transmission electron microscope (TEM). The obtained SEM micrographs of MRSA cells were shown in Figure 6. As shown in Figures 6(a) and 6(b), untreated MRSA cells were typically round-shaped, with smooth and intact cell walls. However, after exposure to AgNPs for 16 h, the cell walls became wrinkled and damaged, indicating the disruption and disorganization of membrane (Figures 6(a1) and 6(b1)). Further, to investigate the ultrastructural changes caused by AgNPs, we performed TEM in order to find additional evidence of the different effect seen. The untreated MRSA cell showed normal cell characteristics such as smooth and intact cell wall showing a well-preserved thick peptidoglycan layer (Figure 7(a)). However, significant ultrastructural changes were observed in cell treated with the AgNPs. Treated* S. aureus *cell exhibited extensive injury and complete disintegration of cell wall and membrane (Figures 7(b) and 7(c)). Large translucent zones have also been seen in the cytoplasm, due to either localized or complete separation of the cell membrane from the cell wall (Figure 7(c)).

Although the exact mode of action of AgNPs to bacterial system is still not very clear. Though, in the literature, various mechanisms have been proposed for the antibacterial effect of AgNPs. In a study, it has been reported that the main cause of bacterial cell death by green synthesized AgNPs was due to the disorganization of cytoplasmic membrane, followed by the leakage of various biomolecules. They also investigated that inhibition of essential enzymes function may also lead to death of bacterial cells [34]. Krishnaraj et al. [35] reported that the change in membrane permeability and loss of integrity of the membrane caused by the action of AgNPs and release of cellular components present inside lead to the bacterial cells death [36]. In the present study, we hypothesized that the positively charged AgNPs may interact with the negatively charged teichoic acid of* S. aureus* cell membrane by electrostatic attraction that causes an increase in membrane permeability and eventually rupture and leakage of intracellular components [37, 38]. Disruption, disorganization, and disintegration of* S. aureus* cell membrane (as supported by our SEM and TEM analysis) might be the other possible mode of AgNPs action [38, 39]. The leakage of intracellular cytoplasmic components due to the loss of membrane integrity may cause shrinkage of the cell membrane that ultimately lead to the lysis of* S. aureus* cell as justified by our TEM micrographs. Das et al. [31] demonstrated that AgNPs synthesized from leaf extract of* Ocimum gratissimum* destroy multidrug resistant bacteria (MRSA) by damaging cell membrane and the production of reactive oxygen species may also contribute to bacterial cell death.

## 4. Conclusion

Ecofriendly and cost effective green method has been employed to synthesize the AgNPs using waste product, i.e., aqueous seed extract of* Phoenix dactylifera* as reducing and stabilizing agents, which is a novel approach to utilize the waste material of date palm for the synthesis nanoparticles. The results obtained clearly showed that AgNPs inhibit growth of MRSA in a dose dependent manner. The strong antibacterial activity of AgNPs at very low concentration against Methicillin-resistant* S. aureus* strains suggests that AgNPs could be an alternative approach for the treatment of medical devices associated infections caused by drug resistant strains.

## Figures and Tables

**Table 1:** Advantage of the green routes of synthesis of AgNPs by *Phoenix dactylifera* over biological, physical, and chemical methods.

**Green method**	**Biological method** **(Microorganism)**	**Physical method**	**Chemical method**
Safe, clean and one step	Simple and easy	Complex and time consuming	Complex and require vigorous processing

Do not require high pressure, energy and temperature	Do not require high pressure, energy and temperature	High energy, pressure and temperature required	Require high temperature, toxic and potentially hazardous chemicals

Eco-friendly, pollution free, biocompatible and highly stable	Eco-friendly and stable	Produced hazardous by-products and pollution	Produced hazardous by-products and pollution

Fast, cost effective and sustainable	Slow but Cost effective	Required expensive and highly complex equipment	Require costly chemicals and solvents

Natural phytochemicals present in plant extracts act as reducing and stabilizing agent	Chemical reducing and stabilizing agent does not required	Chemical reducing and stabilizing agent required	Toxic reducing chemicals and stabilizing agents required i.e., hydrazine, sodium borohydride

Require very low maintenance	Isolation, culturing of microorganism are time consuming and require more maintenance	Require high maintenance	Require high maintenance

## Data Availability

The data used to support the findings of this study are available from the corresponding author upon request.
